# P-2097. Trends in Antibiotic Resistance Among Clinical Isolates in Rural Tamil Nadu: A Prospective Observational Study

**DOI:** 10.1093/ofid/ofaf695.2261

**Published:** 2026-01-11

**Authors:** Suhail Hassan Jalal, Roshni Murali, Suresh Kumar Dorairajan, Giris Sharma, Eiphan Kezia

**Affiliations:** The Tamil Nadu Dr. M.G.R. Medical University, Chennai, Tamil Nadu, India; The Tamil Nadu Dr. M.G.R. Medical University, Chennai, Tamil Nadu, India; The Madras Medical Mission Hospital, Chennai, Tamil Nadu, India; The Tamil Nadu Dr. M.G.R. Medical University, Chennai, Tamil Nadu, India; Madras Medical Mission, Chennai, Tamil Nadu, India

## Abstract

**Background:**

Antimicrobial resistance (AMR) is a major public health concern, particularly in low-resource rural settings with limited antimicrobial stewardship. Rural Tamil Nadu represents a critical region to monitor resistance patterns due to major OTC antibiotic misuse This study assessed antibiotic resistance profiles of bacterial isolates from clinical specimens in rural Tamil Nadu, India.

**Methods:**

A prospective observational study was conducted from October 2024 to February 2025 across three rural primary health centers in Tamil Nadu, India. Clinical specimens (urine, sputum, blood) were processed using standard microbiological methods. Bacterial identification and antibiotic susceptibility testing (Kirby-Bauer disk diffusion) were performed. Resistance trends were stratified by specimen type and organism.

**Results:**

A total of 952 clinical isolates were recovered from 1,229 specimens. The most common organisms: *Escherichia coli* (32.7%), *Klebsiella pneumoniae* (18.4%), *Staphylococcus aureus* (15.3%), *Pseudomonas aeruginosa* (11.7%), and *Enterococcus* spp. (7.2%).

• *E. coli* showed high resistance to ciprofloxacin (78%), co-trimoxazole (64%), and third-generation cephalosporins (ceftriaxone 71%; cefotaxime 68%) with lower resistance to nitrofurantoin (22%) and meropenem (9%).

• *K. pneumoniae* showed resistance to cefotaxime (76%), gentamicin (52%), and amoxicillin-clavulanate (65%); 18% were carbapenem-resistant.

• MRSA prevalence was 39% with 100% susceptibility to vancomycin and linezolid.

• *P. aeruginosa* demonstrated multi-drug resistance (resistance to ≥3 classes) in 27% of isolates, retaining susceptibility to amikacin (81%) and piperacillin-tazobactam (74%).

• *Enterococcus* spp. showed 100% linezolid susceptibility with ampicillin (41%) and vancomycin (5%) resistance.

**Conclusion:**

Alarming resistance rates were observed against first-line antibiotics, including emerging carbapenem resistance in *Klebsiella* spp. and *E. coli* (indicative of ESBL/carbapenemase production). MRSA and vancomycin-resistant *Enterococcus* highlight urgent needs for local stewardship programs. These findings can guide empirical therapy and inform regional antibiotic guidelines to preserve antimicrobial efficacy in resource-limited rural settings.

**Disclosures:**

All Authors: No reported disclosures

